# Associations between polyfluoroalkyl substance and organophosphate flame retardant exposures and telomere length in a cohort of women firefighters and office workers in San Francisco

**DOI:** 10.1186/s12940-021-00778-z

**Published:** 2021-08-28

**Authors:** Cassidy Clarity, Jessica Trowbridge, Roy Gerona, Katherine Ona, Michael McMaster, Vincent Bessonneau, Ruthann Rudel, Heather Buren, Rachel Morello-Frosch

**Affiliations:** 1 Department of Environmental Science, Policy and Management University of California, 130 Mulford Hall, 94720, Berkeley, CA USA; 2 School of Public Health, University of California, Berkeley, CA USA; 3 Department of Obstetrics, Clinical Toxicology and Environmental Biomonitoring Lab, Gynecology and Reproductive Sciences, University of California, San Francisco, CA USA; 4 Department of Cell and Tissue Biology, University of California, San Francisco, CA USA; 5 Department of Obstetrics, Center for Reproductive Sciences, Gynecology and Reproductive Sciences, University of California, San Francisco, CA USA; 6 Silent Spring Institute, Newton, MA USA; 7 Univ. Rennes, Inserm, EHESP, Irset (Institut de Recherche en Santé, Environnement et Travail) – UMR_S 1085, F-35000 Rennes, France; 8 United Fire Service Women, San Francisco, CA USA

**Keywords:** Telomeres, Polyfluoroalkyl substances, Organophosphate flame retardants, Firefighters

## Abstract

**Background:**

Environmental chemical exposures can affect telomere length, which in turn has been associated with adverse health outcomes including cancer. Firefighters are occupationally exposed to many hazardous chemicals and have higher rates of certain cancers. As a potential biomarker of effect, we assessed associations between chemical exposures and telomere length in women firefighters and office workers from San Francisco, CA.

**Methods:**

We measured serum concentrations of polyfluoroalkyl substances (PFAS), urinary metabolites of flame retardants, including organophosphate flame retardants (OPFRs), and telomere length in peripheral blood leukocytes in women firefighters (*N* = 84) and office workers (*N* = 79) who participated in the 2014–15 Women Workers Biomonitoring Collaborative. Multiple linear regression models were used to assess associations between chemical exposures and telomere length.

**Results:**

Regression results revealed significant positive associations between perfluorooctanoic acid (PFOA) and telomere length and perfluorooctanesulfonic acid (PFOS) and telomere length among the whole cohort. Models stratified by occupation showed stronger and more significant associations among firefighters as compared to office workers. Among firefighters in models adjusted for age, we found positive associations between telomere length and log-transformed PFOA (*β* (95%CI) = 0.57(0.12, 1.02)), PFOS (0.44 (0.05, 0.83)), and perfluorodecanoic acid (PFDA) (0.43 (0.02, 0.84)). Modeling PFAS as categories of exposure showed significant associations between perfluorononanoic acid (PFNA) and telomere length among firefighters. Significant associations between OPFR metabolites and telomere length were seen for bis (1,3-dichloro-2-propyl) phosphate (BDCPP) and telomere length among office workers (0.21(0.03, 0.40)) and bis (2-chloroethyl) phosphate (BCEP) and telomere length among firefighters (− 0.14(− 0.28, − 0.01)). For OPFRs, the difference in the direction of effect by occupational group may be due to the disparate detection frequencies and concentrations of exposure between the two groups and/or potential unmeasured confounding.

**Conclusion:**

Our findings suggest positive associations between PFAS and telomere length in women workers, with larger effects seen among firefighters as compared to office workers. The OPFR metabolites BDCPP and BCEP are also associated with telomere length in firefighters and office workers. Associations between chemical exposures and telomere length reported here and by others suggest mechanisms by which these chemicals may affect carcinogenesis and other adverse health outcomes.

**Supplementary Information:**

The online version contains supplementary material available at 10.1186/s12940-021-00778-z.

## Background

The International Agency for Research on Cancer (IARC) has designated the profession of firefighting as “possibly carcinogenic” (Group 2B) [1]. Previous studies indicate that first responders and firefighters have elevated risk of various cancers including: brain, kidney, thyroid, breast, gastro-intestinal, bladder, testicular, prostate, melanoma, lymphomas, and multiple myeloma [2–10]. However, most of these studies have been conducted almost exclusively on men.

There is limited research on female firefighters, despite mounting concern about breast and reproductive cancer risks among this population. This data gap is likely due to the underrepresentation of women in the fire service, however, female membership is increasing, especially in urban areas like San Francisco, which has the highest proportion of women firefighters in the US (15%) [11–13]. Research on female firefighters from Daniels and colleagues showed a non-significant increase in breast cancer incidence and mortality compared to the general US population [2], while research on female firefighters in Florida has found significant increased incidence of Hodgkin’s lymphoma, thyroid cancer, cervical cancer, and brain cancer [8, 14].

In addition to studying cancer, researchers have begun to examine associations between exposures to environmental chemicals and biomarkers of effect with potential relevance for cancer, including telomere length (TL) [15–19]. Telomeres are complexes of repetitive DNA sequences and proteins that cap the ends of chromosomes to protect against degradation and fusion during cell division [20, 21]. Due to incomplete DNA replication at the terminus of DNA strands, telomeres shorten with each cell division. This attrition may be offset by the enzyme telomerase, which restores telomeric DNA [22–24]. Though TL is dynamic, most cells experience net telomere shortening over the life course, eventually triggering cell senescence or apoptosis [24, 25]. Consequently, human TL is negatively associated with age [26–29].

Shortened telomeres have been associated with many diseases, including certain cancers [30–36]. However, telomere lengthening has also been associated with cancer [37–39], and there is evidence of an association between telomere lengthening and breast cancer [40–43]. Though the exact link between TL and cancer remains unclear, research suggests mechanisms by which lengthening or shortening may contribute to carcinogenesis. For instance, telomere shortening may increase genetic instability while telomere lengthening may promote deleterious cell survival and proliferation [25, 44–46].

Firefighters are occupationally exposed to many health-hazardous chemicals, including carcinogens, through activities such as fire suppression and salvage and overhaul at fire scenes [47–53]. Firefighters are also exposed to hazardous chemicals in fire station dust, diesel exhaust, firefighting foams, contaminated fire equipment, and certain firefighting gear [54–59]. Studies have documented firefighters’ exposure to benzene, polycyclic aromatic hydrocarbons (PAHs), formaldehyde, dioxins, polybrominated diphenyl ethers (PBDEs), polyfluoroalkyl substances (PFAS), and organophosphate flame retardants (OPFRs) [60–68].

PFAS, which are widely used for their ability to impart grease, stain, and water resistance to items such as food packaging, non-stick cookware, paints, fabrics, carpets, and furniture [69], are of particular concern for firefighters. Firefighters are exposed to PFAS through the combustion of PFAS-containing products such as furniture and carpet and through firefighting gear and firefighting foams that contain these compounds [57, 64, 70, 71]. Indeed, research shows that firefighters have elevated concentrations of certain PFAS relative to non-firefighters [64, 67, 71]. PFAS exposures have been associated with adverse health outcomes, including cancer [72–79].

Firefighters are also occupationally exposed to flame retardants [65, 68, 80]. While use of polybrominated diphenyl ether (PBDE) flame retardants in consumer products has been gradually phased out due to their toxicity to humans, persistence in the environment, and ability to bioaccumulate [81], OPFRs and other halogenated flame retardants have emerged as replacements and have been found in fire stations [82–84]. There are few epidemiological studies on the human health effects of OPFRs. The existing literature shows associations between OPFR levels in house dust and urine and decreased sperm quality and hormone dysfunction in men [85], and lower thyroxine levels in women with higher urinary levels of diphenyl phosphate [86]. In experimental studies, OPFRs cause endocrine disruptions in sex hormones and thyroid hormones [75, 87–91].

Experimental studies on chemical exposures and telomere length are limited and findings are inconsistent [92]. Previous epidemiological studies have found PFAS exposures are associated with altered TL in humans; perfluorooctanoic acid (PFOA) has been associated with telomere shortening [18] and perfluorooctane sulfonic acid (PFOS) has been associated with telomere lengthening [17]. To our knowledge, no human studies have examined associations between OPFRs and TL, although one study of chemically similar organophosphate insecticides found associations with altered TL, with the direction of effect depending on the insecticide in question [93]. As a possible intermediary between exposure and disease, TL serves as a biomarker of effect for assessing the potential impacts of environmental exposures on human health.

To better characterize firefighters’ exposures with relevance to women’s health outcomes, a collaboration of firefighters, scientists, and environmental health advocates created a community-based participatory research project, the Women Workers Biomonitoring Collaborative (WWBC). We interviewed female firefighters and office workers in San Francisco, CA, and collected biospecimens (urine and serum). Samples were analyzed for PFAS (serum), flame retardant metabolites (urine), and telomere length (whole blood leukocytes). We then assessed the relationship between PFAS and flame retardant metabolite concentrations and TL in female firefighters and office workers.

## Methods

### Recruitment and consent

The WWBC recruitment, enrollment, and sample collection protocol has been described previously [67]. Briefly, recruitment and sample collection took place between June 2014 and March 2015. Firefighter study partners from the San Francisco Fire Department (SFFD) and researchers collaborated on recruitment of both firefighters from SFFD and office worker participants from the City and County of San Francisco. Study inclusion criteria included self-identifying as female, being over 18 years of age, full-time employment, and being a nonsmoker. Additionally, firefighters were required to have at least 5 years of service with the SFFD and to be on “active duty” (i.e., assigned to a fire station) at the time of recruitment. Informed consent was obtained from all participants prior to data collection activities following protocols approved by the Institutional Review Board of the University of California, Berkeley (#2013–07- 5512).

### Data collection and sample processing

Each participant completed an hour-long exposure assessment interview that captured demographics, basic health information, and possible sources of chemical exposure from occupational activities, consumer product use, and diet. A certified phlebotomist collected blood samples in 10 mL additive-free glass tubes and 10 mL EDTA glass tubes. Urine was collected in 60 mL polypropylene specimen cups. All samples were transported in a cooler with ice and processed within 3 h of collection. The serum was separated by allowing clotting at room temperature followed by centrifuging at 3000 rpm for 10 min. The serum and whole blood were aliquoted into 1.2 mL cryovials and urine into 3.5 mL cryovials and stored at − 80 °C until analysis. All samples were processed and analyzed at the University of California, San Francisco.

### PFAS analysis

As described previously [67], twelve PFAS were selected for targeted analysis in serum: perfluorobutanoic acid (PFBA), perfluorohexanoic acid (PFHxA), perfluoroheptanoic acid (PFHpA), perfluorooctanoic acid (PFOA), perfluorononanoic acid (PFNA), perfluorodecanoic acid (PFDA), perfluoroundecanoic acid (PFUnDA), perfluorododecanoic acid (PFDoA), perfluorobutane sulfonic acid (PFBuS), perfluorohexane sulfonic acid (PFHxS), perfluorooctane sulfonic acid (PFOS), and perfluorooctane sulfonamide (PFOSA). The 12 PFAS were analyzed in 0.5 mL of serum using liquid chromatography-tandem mass spectrometry (LC-MS/MS). An Agilent LC1260 (Sta. Clara, CA)- AB Sciex API 5500 (Foster City, CA) platform was used in the analysis. Each sample was prepared for analysis by solid phase extraction using a Waters Oasis HLB cartridge (10 mg, 1 cc). Extracted aliquots of each sample (25 uL) were run in duplicates. The 12 analytes were separated by elution gradient chromatography using Phenomenex Kinetex C18 column (100 × 4.6 mm, 2.6 μ) at 40 °C. Electrospray ionization (negative mode) was used as method of ionization for individual analytes.

Analytes were detected in each sample by multiple reaction monitoring using two transitions per analyte. To determine the presence of each analyte, retention time matching (within 0.15 min) along with the peak area ratio between its qualifier and quantifier ions (within 20%) were used. Quantification of each detected analyte was done by isotope dilution method using a 10-point calibration curve (0.02–50 ng/mL) and employing two C13-labelled PFAS isotopologues. Procedural quality control materials and procedural blanks were run along with the calibration curve at the start, middle, and end of each run. Two QC materials were used at low and high concentrations. To accept the results of a batch run, QC materials measurements must be within 20% of their target values and the precision of their measurements within 20% CV (coefficient of variation).

The limits of quantification for the 12 analytes range from 0.05 to 0.1 ng/mL. Analyte identification from total ion chromatograms was evaluated using AB Sciex Analyst v2.1 software while quantification of each analyte was processed using AB Sciex MultiQuant v2.02 software. Analysts were blinded to firefighter and office worker status of the serum samples during the analysis. Results were reported in ng/mL for all 170 study participants [67].

### Flame retardant analysis

We quantified metabolites of six OPFR chemicals in urine: bis (1,3-dichloro-2-propyl) phosphate (BDCPP), bis (2-chloroethyl) phosphate (BCEP), dibutyl phosphate (DBuP), dibenzyl phosphate (DBzP), di-p-cresyl phosphate (DpCP), di-o-cresyl phosphate (DoCP), and 4 brominated flame retardants: 2,3,4,5-tetrabromobenzoic acid (TBBA), tetrabromobisphenol a (TBBPA), 5-OH-BDE 47, and 5-OH-BDE 100.

Quantitative analysis was performed using liquid-chromatography-tandem mass spectrometry (LC-MS/MS) on an Agilent LC 1260 (Agilent Technologies, Sta. Clara, CA)- AB Sciex 5500 system (Sciex, Redwood City, CA). Freshly thawed urine specimens (1 mL) were deconjugated prior to LC-MS/MS analysis by addition of 450 U *H. pomatia* glucuronidase (Sigma-Aldrich, St Louis, MO) and incubated at 37 °C for two hours with constant shaking. Deconjugated urine samples were prepared for LC-MS/MS analysis by solid phase extraction (SPE) using Waters Oasis WAX cartridges (10 mg, 30 μm, 1 cc). Analytes in the extracted aliquots were separated by elution gradient chromatography using an Agilent ZORBAX Eclipse XDB-C8 column (2.1 × 100 mm, 3.5um) maintained at 50 °C. Negative mode electrospray ionization (ESI) was used to ionize analytes and mass scanning was performed by multiple reaction monitoring. Each analyte was monitored using two transitions and retention time. Quantitation of each analyte was performed by isotope dilution method with their deuterated or C-13 isotopologues as internal standards.

Each sample was injected in duplicate. Procedural quality control materials and procedural blanks were run along with the calibration curve at the start, middle, and end of each run. Two QC materials were used at low and high concentrations. To accept the results of a batch run, QC materials measurements must be within 20% of their target values and the precision of their measurements have ≤20% CV (coefficient of variation). Analyte identification from total ion chromatograms was evaluated using AB Sciex Analyst v2.1 software while quantification of each analyte was processed using AB Sciex MultiQuant v2.02 software. Analysts were blinded to firefighter and office worker status of the urine samples during the analysis.

For both PFAS and flame retardant analyses, each sample extract was injected twice. Results for each sample were accepted if the difference between the average and each value did not exceed 20% of the average. See Additional File 5 for detailed information on PFAS and flame retardant methods.

### Telomere analysis

DNA was extracted from 200 μL of whole blood using the Qiagen Qamp Mini Blood Kit (cat. No. 51104) according to the manufacturer’s instructions. One microgram of DNA from the samples was digested with Hinfl and RsaI, run on 0.8% TAE gels for 6 h and Southern transferred to Nylon membranes. The membranes were hybridized with digoxigenin-labeled telomere probes (Sigma TeloTAGGG Telomere Length Assay, cat. No. 12209136001) followed by incubation with anti-digoxigenin alkaline phosphatase conjugates. DNA bands were detected using chemilunescence and analyzed using ImageQuant software (GE Healthcare). The mean terminal restriction fragment (TRF) length was derived from standards provided in the kit. Results were reported in kilobase pairs (kbp) for 163 participants.

### Statistical analysis

All chemical distributions were skewed and log-transformed using natural logarithms to improve normality. Descriptive statistics such as geometric mean (GM), geometric standard deviation (GSD), and 95% confidence intervals (CI) were calculated for TL, PFAS, and flame retardants with ≥50% detection frequency in at least one occupational group [94, 95].

To assess the relationship between chemical exposure and TL, we developed linear regression models for each compound. Chemical congeners were correlated with each other (Additional file 1) and were run in separate models. Chemicals with ≥70% detection frequency were included in linear models as continuous predictor variables. For models using continuous PFAS concentrations as a predictor, values below the limit of detection (LOD) were substituted with LOD/√2. For models using continuous flame retardant metabolite concentrations as a predictor, we included all LC-MS/MS reported values, including those reported below the LOD, and substituted LOD/√2 for any remaining non-detected values.

For chemicals with < 70% detection frequency but ≥40% detection frequency, chemical concentrations were categorized as either <LOD, LOD-50th%, and > 50th% or as <LOD, ≥LOD, depending on detection frequencies. Telomere data were roughly normally distributed and were therefore not log-transformed.

Potential confounders were selected a priori based on results from previous literature and prior analyses performed on this data [67]. Covariates assessed include demographic variables such as race/ethnicity and education; health variables such as body mass index (BMI), stress, and sleep metrics; and food frequency variables. Spearman correlations were used to test independent relationships between continuous covariates and telomere length and chemical predictors using the Benjamini-Hochberg procedure to control for multiple testing [96]. Analysis of Variance (ANOVA) and t-tests or Wilcoxon rank sum tests were performed to assess differences in TL or chemical predictors across categorical and dichotomous covariates, respectively. Covariates were included in final models as confounders when the association was *p* ≤ 0.10 with both TL (the outcome) and at least one chemical congener (the exposure). For PFAS models, ANOVA for nested models and assessment of percent change in coefficients (Δ ≥ 10%) were also used to inform variable selection.

As exploratory analyses revealed disparate effect estimates by occupation for both PFAS and flame retardant metabolites, models were run on the entire study population where possible and also stratified by occupation. The following equation was used to interpret results from models with a continuous log-transformed chemical predictor: (*β* ∗  *ln* (1 + (*x*/100))) ∗ 1000, where x equals the percent change in PFAS exposure and multiplying by 1000 provides a result in base pairs as opposed to kilobase pairs. For categorical models, raw model estimates in kilobase pairs were multiplied by 1000 for results in base pairs. All analyses were performed in R version 3.5.1 and R studio version 1.1.463 [97, 98].

### PFAS models

We developed minimally adjusted models (Model 1) and fully adjusted models (Model 2) to assess the association between continuous log-transformed PFAS (logPFAS) and TL. Due to the well-documented correlation between age and TL [26–29], age was included in all models. Model 1 was adjusted for continuous age in years. Based on covariate tests described above, Model 2 was adjusted for age, occupation, the number of times dairy products were eaten per week, and the number of times eggs were eaten per week. Models were run for the full cohort and stratified by occupation.

Data visualization using locally weighted regression (loess) curves mapped onto bivariate scatter plots of TL and logPFAS suggested potential non-linear relationships that compelled us to run PFAS Model 2 with PFHxS, PFOA, PFOS, PFNA, and PFDA categorized into quartiles. PFUnDA and PFBuS were categorized into tertiles due to low detection frequency.

### Flame retardant models

Few covariates were associated with flame retardant metabolites within this population [68]. Age was included in all models as well as log-transformed creatinine (logCreatinine) to account for differences in urine dilution [99].

Only BDCPP had sufficient detection frequency to model as a continuous variable for both firefighters and office workers. To test if effect estimates of the BDCPP-TL relationship varied significantly by occupation, we added an interaction term to the full BDCPP-TL model.

Among firefighters, BCEP and DBuP had sufficient detection frequency to include in models as continuous variables though these metabolites were also modeled as categorical variables for comparison across occupations. TBBPA and DpCP were analyzed as categorical variables. The firefighter data for BCEP and DBuP were categorized as <LOD, LOD-50th%, and > 50th% since the detection frequencies were greater than 50%, which allowed for categorization into three groups. Office worker data for BCEP and DbuP and all TBBPA and DpCP data were categorized as <LOD and ≥ LOD due to detection frequencies below 50%. Models for these categorized compounds were stratified by occupation.

## Results

In total, 176 participants enrolled in the study. Six participants (three firefighters and three office workers) were dis-enrolled or did not provide biospecimen samples, and seven participants (two firefighters and five office workers) did not have adequate sample to perform the telomere analysis. The final study sample consisted of 84 firefighters and 79 office workers (*N* = 163) (Table 1). Firefighters had longer telomeres than office workers, but were otherwise similar to office workers in age, dairy consumption, and egg consumption. A detailed description of firefighter and office worker differences in the WWBC has been previously reported [67]. In brief, office workers were more often born outside the US, married, worked at the City and County of San Francisco for less time, and had higher educational attainment concentrations compared to firefighters, although firefighters had higher incomes. Race/ethnicity and BMI were similar across groups [67]. These variables were not associated with TL in our population.

**Table 1:** Descriptive statistics for model parameters by occupation

	Full cohort (*N* = 163)	Firefighters (*N* = 84)	Office Workers (*N* = 79)			Full cohort	Fire- fighters	Office workers
	Mean (SD) or GM (GSD)^a^	Mean (SD) or GM (GSD)^a^	Mean (SD) or GM (GSD)^a^	p-value^b^	LOD^c^	DF^d^(%)	DF(%)	DF(%)
**Age (years)**								
	47.93 (8.10)	47.38 (4.58)	48.52 (10.64)	0.23	–	–	–	–
**Dairy consumption (times per week)**								
	14.45 (8.19)	13.98 (7.30)	14.96 (9.07)	0.47	–	–	–	–
**Egg consumption (times per week)**								
	3.71 (1.93)	3.96 (2.00)	3.46 (1.84)	0.23	–	–	–	–
**Telomere Length (mean TRF in kbp**^e^**)**								
	7.90 (1.12)	8.10 (1.09)	7.68 (1.13)	0.01*	–	–	–	–
**PFAS (ng/mL)**								
PFHxS	3.68 (2.79)	4.55 (2.82)	2.94 (2.64)	0.01*	0.02	100	100	100
PFOA	1.16 (1.76)	1.13 (1.70)	1.19 (1.83)	0.44	0.02	100	100	100
PFOS	4.18 (2.08)	4.33 (1.83)	4.03 (2.35)	0.39	0.02	100	100	100
PFNA	0.69 (1.94)	0.77 (1.98)	0.61 (1.87)	0.09	0.05	100	100	100
PFDA	0.26 (2.09)	0.27 (1.77)	0.24 (2.42)	0.31	0.02	99	100	98
PFUnDA	0.18 (4.44)	0.23 (3.67)	0.14 (5.19)	0.06	0.02	80	87	73
PFBuS	0.13 (4.28)	0.13 (4.19)	0.13 (4.42)	0.85	0.02	73	74	72
**OPFRs (ng/mL)**								
BDCPP	1.92 (5.11)	4.05 (4.59)	0.87 (3.85)	≤0.01*	0.20	95	100	90
BCEP	0.40 (6.11)	0.87 (5.72)	--^f^	--^g^	0.10	60	79	39
DBuP	0.20 (3.92)	0.41 (3.95)	--^f^	--^g^	0.10	56	82	28
TBBPA	--^f^	--^f^	--^f^	--^g^	0.20	44	46	41
DpCP	--^f^	--^f^	--^f^	--^g^	0.10	29	42	16

^a^ Geometric mean (GM) and geometric standard deviation (GSD) computed for egg consumption, all PFAS, and all OPFRs due to skewed distributions

^b^ p-values derived from Wilcoxon rank-sum tests

^c^ LOD = limit of detection

^d^ DF = detection frequency

^e^ kilobase pairs

^f^ GMs were not calculated for chemicals with detection frequencies below 50%

^g^ Wilcoxon rank-sum tests were not performed for chemicals with detection frequencies below 50% in any group

^h^ All summary statistics calculated using LOD/sqrt [2] for those chemicals with less than 100% detection frequency

### PFAS exposure and telomere length

We measured serum for 12 PFAS, four of which (PFBA, PFHxA, PFHpA, and PFOSA) had no measurable concentrations above the LOD in any participant. Seven PFAS congeners had detection frequencies greater than 70%, of which four had detection frequencies of 100% (PFHxS, PFOA, PFOS, PFNA). PFHxS was found at significantly higher concentrations among firefighters compared to office workers. Higher concentrations of PFNA were also observed among firefighters, however the group difference was not statistically significant in this subset of WWBC data. Distributions of the remaining PFAS were similar across groups (Table 1). A full description of differences and predictors of PFAS concentrations in firefighters and office workers is described elsewhere [67].

Of the covariates assessed as potential confounders of the PFAS-TL relationship, only age, occupation, dairy consumption, and egg consumption met our criteria for inclusion in fully adjusted models. Effect estimates were generally larger among firefighters compared to office workers (Table 2 and Additional file 2). In both models, exposure to PFOA and PFOS was associated with significantly longer TL among the entire cohort. In Model 1, a doubling (or 100% increase) of PFOA concentration was associated with a 273 (95% CI 54, 493) base pair (bp) increase in TL. In Model 2, a doubling in PFOA was associated with a 240 (95% CI 25, 455) bp increase in TL. A doubling in PFOS concentration was associated with a 183 (95% CI 15, 352) bp increase in TL in Model 1, and a 172 (95% CI 5, 340) bp increase in TL in Model 2 (Table 2).

**Table 2:** Estimated base pair change in telomere length for a doubling of PFAS concentration

	Full Cohort Δbp^**c**^ (CI^d^)	Firefighters Δbp (CI)	Office Workers Δbp (CI)
**PFHxS**			
Model 1^a^	103 (−16,223)	72 (−92,235)	88 (−98,275)
Model 2^b^	84 (− 35,202)	91 (−69,252)	69 (−116,254)
**PFOA**			
Model 1	273 (54,493)*	395 (85,705)*	175 (−135,485)
Model 2	240 (25,455)*	329 (13,645)*	165 (−140,470)
**PFOS**			
Model 1	183 (15,352)*	304 (33,576)*	91 (− 125,308)
Model 2	172 (5340)*	272 (− 4548)	124 (−95,342)
**PFNA**			
Model 1	126 (−60,312)	103 (− 144,350)	65 (− 233,362)
Model 2	68 (−117,253)	52 (−195,299)	116 (−183,416)
**PFDA**			
Model 1	137 (−27,302)	300 (16,585)*	34 (−170,239)
Model 2	104 (−58,266)	255 (−31,541)	60 (−148,267)
**PFUnDA**			
Model 1	26 (−55,106)	79 (−50,209)	−31 (−136,75)
Model 2	13 (−67,93)	61 (−67,190)	2 (−114,117)
**PFBuS**			
Model 1	10 (−72,92)	−33 (− 151,84)	50 (−67,168)
Model 2	23 (−58,104)	−4 (−123,114)	46 (− 72,165)

^a^ Model 1 adjusted for age (years)

^b^ Model 2 adjusted for age (years), dairy and egg consumption (times per week), and occupation (in full group only)

^c^ change in base pairs

^d^ CI = 95% confidence interval

* statistically significant (*p* ≤ 0.05)

Among firefighters, exposure to PFOA, PFOS, and PFDA was significantly associated with longer TL in Model 1. In Model 2 (adjusted for age, dairy consumption, and egg consumption), only PFOA remained significantly associated with TL. Among firefighters, a doubling of PFOA concentration was associated with a 395 (95% CI 85,705) base pair (bp) increase in TL in Model 1, and a 329 (95% CI 13,645) bp increase in TL in Model 2. In Model 1, a doubling in firefighters’ PFOS concentration was associated with a 304 (95% CI 33,576) bp increase in TL, and a doubling in firefighters’ PFDA concentration is associated with a 300 (95% CI 16,585) bp increase in TL.

Most PFAS were positively associated with TL in office workers, though effect estimates were smaller than for firefighters and none were statistically significant. No interaction terms testing for effect modification by occupation were statistically significant.

**Fig. 1:**
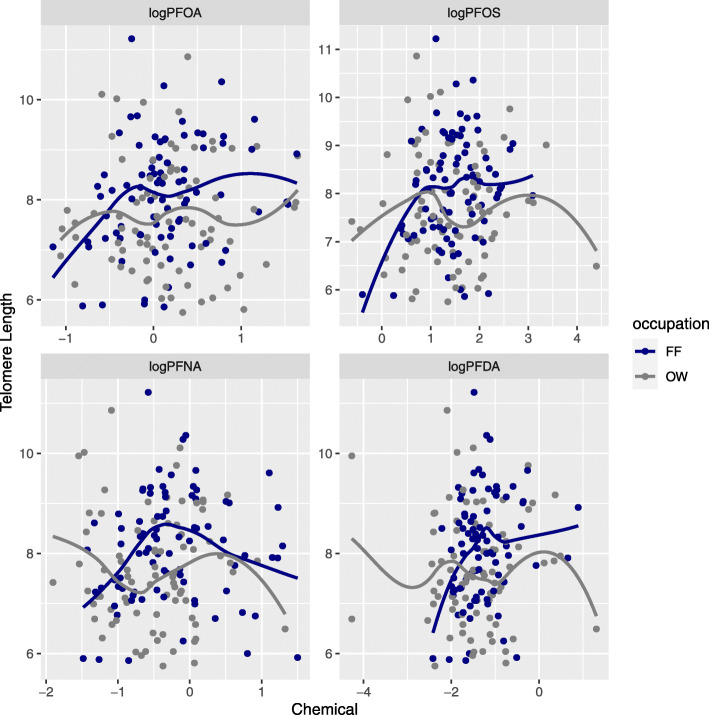
Loess curves^a^ for telomere length and log-transformed PFAS by occupation. ^a^ Locally-weighted regression with span = 0.75. OW=office worker; FF=firefighter

To assess the shape of the exposure-response relationships, we modeled locally weighted regression (loess) curves atop unadjusted scatter plots of TL and logPFAS, stratified by occupation. Among firefighters, the loess curves suggested potential non-linear exposure-response relationships, with log-transformed PFOA, PFOS, PFNA, and PFDA exhibiting a somewhat conserved pattern (Fig. 1). In firefighters, exposure to these four PFAS compounds appears to be associated with increasing TL from low to intermediate concentrations and unchanging or decreasing TL at higher concentrations.

To further explore this relationship, we included PFAS as categorical variables in fully adjusted models. Table 3 details the estimated base pair change for a categorical increase of PFAS exposure relative to the referent (first quartile) from fully adjusted models. Among firefighters, PFNA, which had non-statistically significant associations with TL in the continuous linear Models 1 and 2, had significant effect estimates for each quartile of exposure relative to the referent, with the greatest increase in the second quartile as was suggested by the loess curve.

**Table 3:** Estimated^a^ base pair change in telomere length relative to the reference group

	Firefighters Δbp^b^ (CI^c^)	Office Workers Δbp (CI)
**PFHxS**		
Reference^d^	–	–
Q2	−62 (− 728, 604)	209 (− 515, 933)
Q3	− 180 (− 850, 491)	238 (− 522, 998)
Q4	366 (− 301, 1033)	163 (− 563, 889)
**PFOA**		
Reference	–	–
Q2	209 (− 464, 882)	10 (− 704, 724)
Q3	290 (− 387, 967)	500 (−197, 1198)
Q4	368 (− 327, 1063)	143 (− 584, 869)
**PFOS**		
Reference	–	–
Q2	211 (− 453, 876)	301 (− 417, 1019)
Q3	599 (−82, 1280)	−18 (− 716, 681)
Q4	397 (− 288, 1082)	426 (− 357, 1210)
**PFNA**		
Reference	–	–
Q2	1250 (631, 1869)*	− 344 (− 1064, 376)
Q3	809 (197, 1421)*	− 46 (− 769, 676)
Q4	668 (29, 1307)*	164 (−576, 904)
**PFDA**		
Reference	–	–
Q2	187 (− 472, 845)	−4 (− 729, 721)
Q3	614 (−48, 1275)	−6 (− 722, 710)
Q4	396 (−265, 1058)	245 (− 489, 978)
**PFUnDA**		
Reference	–	–
T2	22 (− 549, 592)	− 154 (− 774, 466)
T3	488 (−88, 1065)	215 (− 430, 861)
**PFBuS**		
Reference	–	–
T2	−184 (− 782, 414)	144 (− 503, 791)
T3	−19 (− 606, 569)	15 (− 598, 628)

^a^ Model adjusted for age (years) and dairy and egg consumption (times per week)

^b^ change in base pairs

^c^ CI = 95% confidence interval

^d^ Reference category is first quartile or tertile of exposure

* Statistically significant (*p* ≤ 0.05)

### Flame retardant exposure and telomere length

We measured 10 flame retardant metabolites in urine, two of which (5-OH-BDE 47 and 5-OH-BDE 100) had no concentrations above the LOD. Descriptive statistics of flame retardant data revealed disparate distributions of chemical concentrations between occupational groups, with firefighters’ concentrations measured at higher detection frequencies and higher concentrations relative to office workers (Table 1 and Additional file 4). A more in-depth description of the differences in flame retardant concentrations between occupational groups and associated covariates is discussed in Trowbridge et al., 2020 [68]. In brief, BDCPP, BCEP, DBuP, and DpCP were all measured at significantly higher concentrations among firefighters compared to office workers (Table 1 and Additional file 4). Though it had an overall detection frequency of only 29%, DpCP was modeled as a categorical exposure variable because it had a detection frequency of 42% among firefighters. BDCPP was the only flame retardant metabolite with sufficient detection frequency (≥ 70%) to include in models as a continuous variable for both firefighters and office workers. BCEP and DBuP had ≥70% detection frequency among firefighters so were included in firefighter models as continuous variables.

Table 4 and Additional file 3 show results from stratified linear models controlling for age and logCreatinine. BDCPP concentrations were negatively associated with TL in firefighters and positively associated with TL in office workers. The effect in office workers was statistically significant, with a doubling in BDCPP concentration associated with a 148 (95% CI 22, 274) bp increase in TL. An interaction term for BDCPP and occupation was significant, suggesting that the BDCPP-TL relationship differs significantly by occupation (*p*-value≤0.01). In models for BCEP, increasing concentration was significantly associated with decreasing TL, with a doubling in BCEP associated with a 99 (95% CI − 194, − 5) bp decrease in TL.

**Table 4:** Estimated^a^ base pair change in telomere length for a doubling in OPFR metabolite concentration

	Firefighter Δbp^b^ (CI^c^)	Office Worker Δbp (CI)
**BDCPP**	−70 (− 184,44)	148 (22,274)*
**BCEP**	−99 (− 194,-5)*	–
**DBuP**	10 (−113,132)	–

^a^ Models adjusted for age (years) and log-transformed creatinine

^b^ change in base pairs

^c^ CI = 95% confidence interval

* statistically significant (p ≤ 0.05)

We also ran models with BCEP, DBuP, TBBPA, and DpCP as categorical variables among both firefighters and office workers (Table 5 and Additional file 3). All models were stratified due to disparate detection frequencies between groups, which precluded running single models for the full cohort, save for BDCPP. In categorical models, BCEP and TBBPA showed similar patterns of association with TL, with negative effect estimates in firefighters and positive effect estimates in office workers, however, these effect estimates were not statistically significant.

**Table 5:** Estimated^a^ base pair change in telomere length relative to the reference group (<LOD)

	Firefighters Δbp^b^ (CI^c^)	Office Workers Δbp (CI)
**BCEP**		
< LOD/Ref^d^	–	–
LOD-50%	− 557 (− 1240, 126)	–
> 50th%	−578 (− 1189, 32)	–
> LOD	–	259 (− 257, 774)
**DBuP**		
< LOD/Ref	–	–
LOD-50%	12 (− 699, 724)	–
> 50%	20 (−646, 687)	–
> LOD	–	134 (− 427, 694)
**TBBPA**		
< LOD/Ref	–	–
> LOD	−227 (−705, 251)	349 (− 160, 858)
**DpCP**		
< LOD/Ref	–	–
> LOD	− 383 (− 868, 101)	−57 (−749, 635)

^a^ Models adjusted for age (years) and log-transformed creatinine

^b^ change in base pairs

^c^ CI = 95% confidence interval

^d^ Categories based on detection frequency. Reference category is <LOD for both firefighters and office workers

## Discussion

This community-based participatory research study examined cross-sectional relationships between PFAS and flame retardant exposures and TL in female firefighters and office workers in San Francisco, CA. To our knowledge, this is the first study to assess the association between chemical exposures and telomere length in female firefighters, and the first study to assess the association between OPFRs and telomere length.

PFAS concentrations among the entire cohort were comparable to US women in the 2013–14 National Health and Nutrition Examination Survey except for PFHxS, which was higher in this study cohort, and PFOA, which was lower in this study cohort. Firefighters had higher concentrations of several PFAS compared to NHANES data [67]. Firefighters also had higher concentrations of BDCPP, BCEP, and DBuP, while office workers had comparable concentrations of BDCPP and lower concentrations of BCEP and DbuP than women in the 2013–14 NHANES [68]. Previously published work on WFBC data further explore predictors of exposure and comparisons to other studies [67, 68].

Our analyses of PFAS data revealed statistically significant positive associations between PFOA and PFOS and telomere length among the full cohort, with larger effect estimates among firefighters. Among firefighters, PFOA, PFOS, PFNA, and PFDA were positively associated with TL. Effect estimates among office workers were mostly positive or null across PFAS. These results suggest that exposure to some PFAS, particularly PFOA, PFOS, PFNA, and PFDA, may be associated with telomere lengthening in female firefighters. Prior studies on PFAS exposure and TL are limited and report mixed results. Huang et al., 2019 examined PFAS and TL in National Health and Nutrition Examination Survey (NHANES) data and reported a strong positive association between PFOS and leukocyte TL in adults and null associations for other PFAS and TL [17]. Vriens et al., 2019 found a negative association between PFOA and leukocyte TL in adults aged 50 to 65 years using multipollutant models [18]. Zota et al., 2018 similarly used multipollutant models and found no significant associations between prenatal PFAS exposure and repeated measures of leukocyte TL in overweight and obese low-income mothers with an average age of 27.9 years [100]. While the literature on the PFAS and TL relationship seems equivocal, such studies may not be comparable due to underlying differences in study populations, methodological approaches, and other confounding and modifying factors.

Our results show that PFAS exposure is associated with telomere lengthening. Previous work has shown that exposure to environmental chemicals is associated with longer TL [16, 19, 101, 102]. Mitro et al., 2016 proposed that certain POPs, particularly some polychlorinated biphenyls (PCBs), activate the aryl hydrocarbon receptor (AhR), which up-regulates telomerase and may therefore promote cancer [19]. Telomerase activation is necessary for cell immortality, which is in turn necessary for tumorigenesis [103]. There is some limited evidence of AhR activation by PFAS [104], and so telomerase activation may play an important role in the PFAS-TL relationship. Where peripheral blood leukocytes are used, telomerase activity may differ by cell type [105]. Experimental research that includes the measurement of these chemicals and telomerase is needed to explore potential pathways.

Results from flame retardant analyses revealed different effects on TL by occupational status, with flame retardant exposure among firefighters associated with a decrease in TL, and exposure among office workers associated with an increase in TL. However, results were statistically significant only for BDCPP and TL in office workers, and BCEP and TL in firefighters. These differences in effects may not be comparable across occupational groups due to the significantly higher exposure concentrations and detection frequencies of flame retardant metabolites in firefighters relative to office workers.

Pending further work to characterize the exposure-response relationship, these findings align with other research that has documented variable impacts on TL by dose of environmental chemicals. For instance, Zhang et al., 2003 showed that low doses of arsenite in vitro promoted telomerase activity, sustained or lengthened telomeres, and increased cell proliferation, while higher doses of arsenite decreased telomerase and telomere length and promoted apoptosis [106]. Similar findings were reported by Ferrario et al., 2009 [107]. Shin et al., 2010 reported an analogous trend with POPs and TL in NHANES data, finding longer TL at lower concentrations of POPs and decreased lengthening as POP concentration increased [101].

In both the PFAS-TL and flame retardant-TL analyses, effect estimates differed by occupation. In the PFAS-TL relationship, the differences were in magnitude and estimates of statistical interaction were not significant. In the flame retardant-TL relationship, the differences were in direction and the estimate of statistical interaction between occupation and BDCPP in the BDCPP-TL relationship was significant. While the effect modification by occupation seen in the flame retardant-TL relationship may be attributable to variable effects by dose, it is also possible that there are unmeasured co-exposures affecting TL.

Firefighters are occupationally exposed to many different chemicals including benzene, PAHs, formaldehyde, dioxins, and PBDEs [60–65]. Effect estimate differences may be due to unmeasured confounding, including unmeasured chemical co-exposures in firefighters that also have an impact on TL. Non-targeted and/or exposomic approaches are required to improve the characterization of exposures to chemical mixtures and their effects on biological response markers, including TL [108–110].

This was a cross-sectional study, which precludes causal inference (Allen, 2017). Exposure misclassification from cross-sectional sampling may be less relevant when analyzing serum concentrations of the PFAS assessed here due to their relatively long half-lives in the body [111–113]. Flame retardant metabolites were measured in single spot urine samples so temporal variability in concentrations could result in exposure misclassification; however, prior studies indicate that there is temporal stability in OPFR metabolite measurements in urine [114, 115]. Furthermore, we accounted for urine dilution by including creatinine measurements in our models. Although specific gravity may be considered a preferable measure of urine dilution [116], a study assessing variability in organophosphate metabolite measurements in urine found that temporal variability of creatinine-adjusted metabolite concentrations was lower than that of specific gravity-adjusted and unadjusted metabolite concentrations [114]. We do not have complete blood count data from participants, elements of which may correlate with telomere length [117]. Not all potentially relevant flame retardants, including diphenyl phosphate (DPhP), were measured in this analysis.

## Conclusion

We found positive associations between PFOA and PFOS and telomere length in women workers, with larger effects seen among firefighters compared to office workers for PFOA, PFOS, PFDA, and PFNA. The OPFR metabolites BDCPP and BCEP may also be associated with altered telomere length in women workers. While further exposomic and mechanistic research is needed to more holistically characterize exposures and confirm their relationships with telomere length, the associations reported here suggest mechanisms by which these chemicals may affect carcinogenesis and other adverse health outcomes.

## Supplementary Information

**Additional file 1.** Correlation matrix for PFAS and FR chemical congeners.

**Additional file 2.** Effect estimates^b^ for PFAS exposure on telomere length by occupation from minimally adjusted and fully adjusted^a^ continuous linear models.

**Additional file 3.** Effect estimates for OPFR exposure on telomere length by occupation from adjusted^a^ linear models.

**Additional file 4. **Flame retardant metabolite concentrations (ng/mL) by occupation; values below the LOD were substituted with LC-MS/MS reported values below the LOD where available, and LOD/√2 for remaining non-detect values; grey lines represent chemical-specific LODs; *p*-values indicate significance of permutation tests assessing difference in concentration distributions between occupational groups.

**Additional file 5: Table S1.** Quantifier and qualifier transitions for the twelve perfluroalkyl substances biomonitored in the study. **Table S2.** Quantifier and qualifier transitions for the ten flame retardants biomonitored in the study.

## Data Availability

The datasets generated and analyzed for this study are not publicly available because they contain personally identifiable information. They may be made available from the corresponding author on reasonable request.

## Abbreviations

WWBC: Women Workers Biomonitoring Collaborative
TL: telomere length
PFAS: polyfluoroalkyl substances
PFOA: perfluorooctanoic acid
PFOS: perfluorooctane sulfonic acid
PFNA: perfluorononanoic acid
PFDA: perfluorodecanoic acid
PFHxS: perfluorohexane sulfonic acid
PFuNDA: perfluoroundecanoic acid
PFBuS: perfluorobutane sulfonic acid
PFBA: perfluorobutanoic acid
PFHxA: perfluorohexanoic acid
PFDoA: perfluorododecanoic acid
PFHpA: perfluoroheptanoic acid
PFOSA: perfluorooctane sulfonamide
OPFR: organophosphate flame retardant
BDCPP: bis (1,3-dichloro-2-propyl) phosphate
BCEP: bis (2-chloroethyl) phosphate
DbuP: dibutyl phosphate
DBzP: dibenzyl phosphate
DpCP: di-p-cresyl phosphate
DoCP: di-o-cresyl phosphate
TBBA: 2,3,4,5-tetrabromobenzoic acid
TBBPA: tetrabromobisphenol a
PAH: polycyclic aromatic hydrocarbon
PBDE: polybrominated diphenyl ether
LC-MS/MS: liquid chromatography-mass spectrometry
GM: geometric mean
GSD: geometric standard deviation
CI: confidence interval
LOD: limit of detection
DF: detection frequency
