# Supplementary figures and images for: Associations between polyfluoroalkyl substance and organophosphate flame retardant exposures and telomere length in a cohort of women firefighters and office workers in San Francisco
Source: Environ Health. 2021 Aug 28;20:97. doi: 10.1186/s12940-021-00778-z (PMC8403436; doi:10.1186/s12940-021-00778-z)

**Additional file 1**

**Correlation matrix for PFAS and FR chemical congeners.**
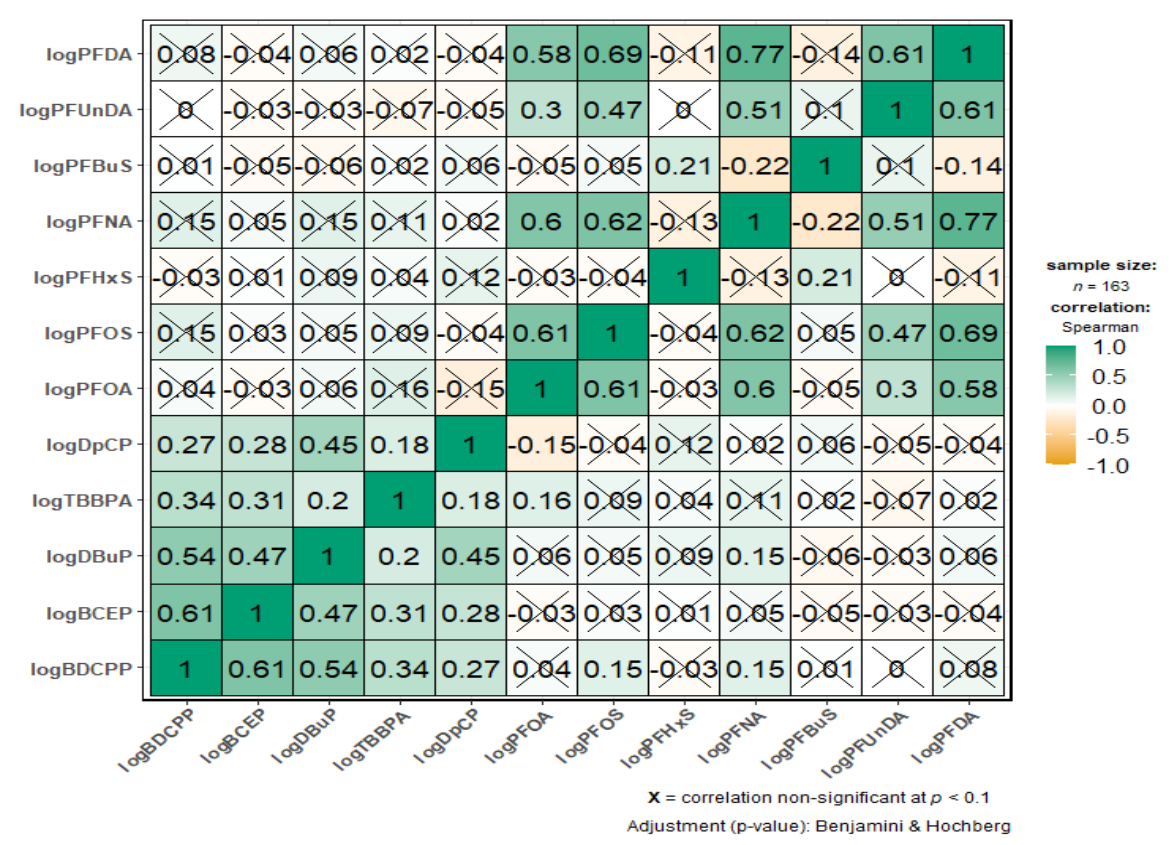

Supplement: Supplementary file 1 — Additional file 1. Correlation matrix for PFAS and FR chemical congeners. [file 12940_2021_778_MOESM1_ESM.docx]
