# Supplementary material for: Associations between polyfluoroalkyl substance and organophosphate flame retardant exposures and telomere length in a cohort of women firefighters and office workers in San Francisco
Source: Environ Health. 2021 Aug 28;20:97. doi: 10.1186/s12940-021-00778-z (PMC8403436; doi:10.1186/s12940-021-00778-z)
Supplement: Supplementary file 4 — Additional file 4. Flame retardant metabolite concentrations (ng/mL) by occupation; values below the LOD were substituted with LC-MS/MS reported values below the LOD where available, and LOD/√2 for remaining non-detect values; grey lines represent chemical-specific LODs; p-values indicate significance of permutation tests assessing difference in concentration distributions between occupational groups. [file 12940_2021_778_MOESM4_ESM.docx]

**Additional file 4**


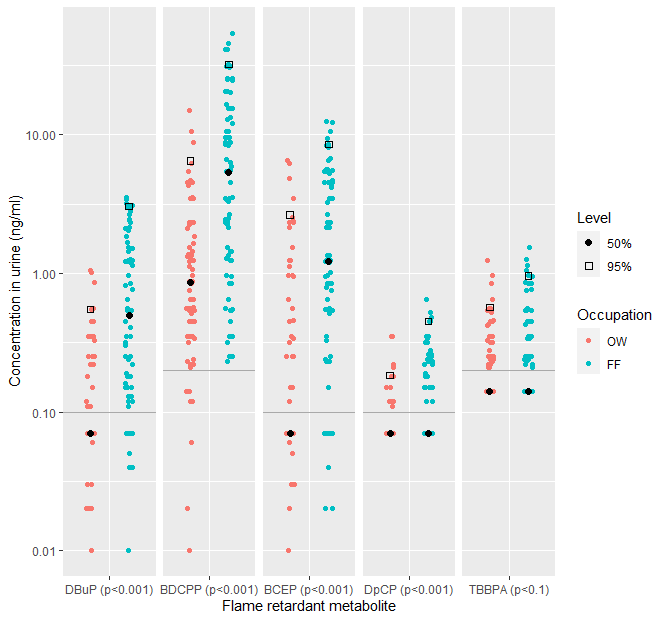


**Flame retardant metabolite concentrations (ng/mL) by occupation**; values below the LOD were substituted with LC-MS/MS reported values below the LOD where available, and LOD/√2 for remaining non-detect values; grey lines represent chemical-specific LODs; p-values indicate significance of permutation tests assessing difference in concentration distributions between occupational groups.
