# Supplementary material for: Associations between polyfluoroalkyl substance and organophosphate flame retardant exposures and telomere length in a cohort of women firefighters and office workers in San Francisco
Source: Environ Health. 2021 Aug 28;20:97. doi: 10.1186/s12940-021-00778-z (PMC8403436; doi:10.1186/s12940-021-00778-z)
Supplement: Supplementary file 5 — Additional file 5: Table S1. Quantifier and qualifier transitions for the twelve perfluroalkyl substances biomonitored in the study. Table S2. Quantifier and qualifier transitions for the ten flame retardants biomonitored in the study. [file 12940_2021_778_MOESM5_ESM.docx]

**Additional File 5**

**Method Details for Perfluoroalkyl Substances (PFAS)**

Solid phase extraction was used in preparing the samples for analysis using a Waters Oasis HLB cartridge (10 mg, 1 cc). The column was conditioned with 1 column volume (CV) each of methanol and 0.1% formic acid in water. After loading the column with the sample, the column was washed consecutively with (1) 1 CV 0.1% formic acid in water, (2) 1CV 0.1% formic acid in 50% methanol, and (3) 1CV ammonium hydroxide in water. The samples were eluted in 1% ammonium hydroxide in methanol. After injection of the sample into the LC-MS/MS, the analytes were separated by elution gradient chromatography using 1% formic acid in water as mobile Phase A (MPA) and acetonitrile as Mobile Phase B (MPB). The gradient used consists of the following: 0-3 min: 20% Mobile Phase A (MPA) to 40% MPA; 3.01-8 min: 20% MPA. The transitions for each analyte monitored during mass spectrometry are detailed in the table below.

Table S1. Quantifier and qualifier transitions for the twelve perfluroalkyl substances biomonitored in the study

| Analyte | Quantifier (m/z) | | Qualifier (m/z) | |
| --- | --- | --- | --- | --- |
|  | Q1 | Q3 | Q1 | Q3 |
| Perfluorobutanoic acid (PFBA) | 213.0 | 169.1 | 213.0 | 99.0 |
| Perfluorohexanoic acid (PFHxA) | 313.0 | 269.0 | 313.0 | 119.0 |
| Perfluoroheptanoic acid (PFHpA) | 362.9 | 318.8 | 362.9 | 168.9 |
| Perfluorooctanoic acid (PFOA) | 412.9 | 368.8 | 412.9 | 169.0 |
| Perfluorononanoic acid (PFNA) | 462.8 | 418.9 | 462.8 | 218.9 |
| Perfluorodecanoic acid (PFDA) | 512.8 | 680.9 | 512.8 | 218.8 |
| Perfluoroundecanoic acid (PFUnDA) | 562.8 | 518.9 | 562.8 | 268.9 |
| Perfluorododecanoic acid (PFDoA) | 612.9 | 568.8 | 612.9 | 169.0 |
| Perfluorobutane sulfonic acid (PFBuS) | 299.0 | 80.0 | 299.0 | 99.0 |
| Perfluorohexane sulfonic acid (PFHxS) | 398.9 | 79.8 | 398.9 | 119.0 |
| Perfluorooctane sulfonic acid (PFOS) | 498.9 | 79.9 | 498.9 | 98.9 |
| Perfluorooctane sulfonamide (PFOSA) | 487.8 | 77.9 | 497.8 | 48.0 |

**Method Details for Flame Retardants**

Solid phase extraction was used in preparing the samples for analysis using a Waters Oasis WAX cartridge (10 mg, 1 cc). The column was prewashed with 5 column volumes (CV) 5% ammonium in methanol before being activated with 1 CV water. After loading the column with the sample, the column was washed with 1 CV 2% formic acid in water in water. The samples were eluted in 5% ammonium hydroxide in methanol. After injection of the sample into the LC-MS/MS, the analytes were separated by elution gradient chromatography using 20 mM ammonium acetate in water as mobile Phase A (MPA) and acetonitrile as Mobile Phase B (MPB). The gradient used consists of the following: 0-0.5 min: 5% MPB; 0.5-7.5 min: gradient 75% MPB; 7.5-9 min: gradient to 100% MPB; 9-11 min: 100% MPB; 11.1-15 min: 5% MPB. The transitions for each analyte monitored during mass spectrometry are detailed in the table below.

Table S2. Quantifier and qualifier transitions for the ten flame retardants biomonitored in the study

| Analyte | Quantifier (m/z) | | Qualifier (m/z) | |
| --- | --- | --- | --- | --- |
|  | Q1 | Q3 | Q1 | Q3 |
| Bis(1,3-dichloro-2-propyl) phosphate (BDCPP) | 318.9 | 141.0 | 318.9 | 59.0 |
| Bis(2-chloroethyl) phosphate (BCEP) | 222.9 | 141.0 | 222.9 | 126.9 |
| Dibutyl phosphate (DBuP) | 209.6 | 78.8 | 209.6 | 153.0 |
| Dibenzyl phosphate (DBzP) | 277.0 | 79.0 | 277.0 | 63.0 |
| Di-o-cresyl phosphate (DoCP) | 276.9 | 107.0 | 276.9 | 169.0 |
| Di-p-cresyl phosphate (DpCP) | 279.0 | 90.9 | 279.0 | 165.7 |
| 2,3,4,5-tetrabromobenzoic acid (TBBA) | 434.7 | 390.7 | 434.7 | 392.7 |
| Tetrabromobisphenol A (TBBPA) | 546.8 | 283.2 | 546.8 | 58.9 |
| 5-OH-BDE 47 | 498.9 | 59.0 | 498.9 | 140.8 |
| 5-OH-BDE 100 | 578.6 | 79.0 | 578.6 | 81.0 |
